# Dacomitinib, an Irreversible Pan-ErbB Inhibitor Significantly Abrogates Growth in Head and Neck Cancer Models That Exhibit Low Response to Cetuximab

**DOI:** 10.1371/journal.pone.0056112

**Published:** 2013-02-06

**Authors:** Ferdows Ather, Habib Hamidi, Marlena S. Fejzo, Stephen Letrent, Richard S. Finn, Fairooz Kabbinavar, Christian Head, Steven G. Wong

**Affiliations:** 1 Department of Medicine, David Geffen School of Medicine, University of California Los Angeles, Los Angeles, California, United States of America; 2 Clinical Development and Medical Affairs, Pfizer Oncology, San Diego, California, United States of America; Dresden University of Technology, Germany

## Abstract

Aberrant epidermal growth factor (EGF) signaling is associated with tumor growth in squamous cell carcinoma of the head and neck in humans (HNSCC), and is a major focus of targeted therapy. Cetuximab, a monoclonal antibody against EGFR, has been successful at prolonging survival but has only a 10% tumor shrinkage response rate in a clinical setting. The goal of this study was to compare dacomitinib (PF-00299804), a next generation small molecule tyrosine kinase inhibitor that irreversibly blocks multiple HER family receptors (HER-1 (EGFR), HER-2 and HER-4 tyrosine kinases), to cetuximab, the current FDA approved anti-EGFR medication for HNSCC and erlotinib, an EGFR specific small molecule tyrosine kinase inhibitor. Dacomitinib, erlotinib and cetuximab were tested in a panel of 27 HNSCC cell lines. Treatment with 100 ug/ml of cetuximab or 1 uM of erlotinib inhibited growth by at least 50% in 7/27 cell lines, while treatment with 1 uM of dacomitinib had similar growth inhibition in 17/27 lines. Cell lines representing three levels of sensitivity to dacomitinib were further examined using Western blots, cell cycle and apoptosis analysis. Treatment with 100 nM of dacomitinib reduced EGFR activity and downstream AKT and ERK pathways more effectively than treatment with 100 ug/ml of cetuximab in all ten tested lines. Although both compounds induced apoptosis at similar levels, dacomitinib caused greater G0/G1 arrest. Sensitivity to EGFR blockade was associated with levels of EGFR and ERK and was not associated with common oncogenic mutations and copy number variations. Phosphorylated and total EGFR and ERK levels correlate with sensitivity to both cetuximab and dacomitinib. Three of the four lines in the exquisitely sensitive group had the highest levels of phosphorylated and total EGFR and ERK among the ten lines selected, while the three resistant lines collectively had the lowest levels. Neither pAKT nor tAKT was associated with sensitivity.

## Introduction

Squamous cell carcinoma of the head and neck (HNSCC), which consists of cancers originating in the oral and nasal cavities, larynx, pharynx, lip and sinuses, is the sixth most common cancer worldwide with an incidence surpassing 500,000 cases annually [1], [2]. Despite the evolving model of multimodality management integrating surgical intervention, chemotherapy, and radiation therapy, overall survival remains poor with a 5-year relative survival rate below 50% (SEER HNSCC stats). Head and neck cancer management holds considerable potential for the utilization of targeted biologic therapies, a strategy which has been making significant advances in the treatment of other histologies including cancers of the breast [3], colon [4], and lung cancer [5]. The primary causative factor for lung and head and neck cancer is smoking, and both possess similar molecular characteristics which have been implicated in the pathogenesis of disease, such as a key role of the human epidermal growth factor receptor (EGFR) in tumor growth.

EGFR, which is highly expressed in a significant majority (up to 80–100%) of HNSCC, is of the prototype receptor of the HER tyrosine kinase receptor family, which includes HER1/ErbB-1/EGFR, HER2/neu/ErbB-2, HER3/ErbB-3 and HER4/ErbB-4 [6]. Binding one of its seven ligands (which includes EGF and TGF-alpha) induces homodimerization and heterodimerization with other family member and phosphorylation at several tyrosine residues in the C-terminal domain [7]. Binding of specific ligand, such as the epidermal growth factor (EGF) and transforming growth factor (TGF-alpha) to EGFR, results in receptor dimerization and initiation of intracellular signaling pathways. Major downstream signaling is via the Ras-Raf-MAPK pathway. Activation of Ras initiates a multistep phosphorylation cascade that leads to the activation of MAPKs, ERK1 and ERK2, which ultimately regulate transcription of molecules involved in cell proliferation [8]. Another important target in EGFR signaling is phosphatidylinositol 3-kinase (P13K) and the downstream protein-serine/threonine kinase Akt. This latter protein kinase transduces molecular signals which trigger crucial steps for cell growth and survival [8], [9].

Aberrant activation of EGFR and its downstream pathways has been implicated in several malignancies [10]. Overexpression of EGFR in HNSCC has been associated with lower response rates to standard chemotherapy, and increased recurrence and resistance to radiation treatment [11], [12], [13]. Several compounds targeting EGFR have successfully entered clinical practice in cancer medicine including small molecules that bind the tyrosine kinase domain of EGFR such as gefitinib [14] (AstraZeneca, lung cancer) and erlotinib [15] (OSI/Genentech, lung and pancreatic cancer) and the monoclonal antibodies cetuximab [16](BMS/Imclone, colorectal, lung and head and neck cancer) and panitumumab [17] (Amgen, colorectal cancer) which bind its extracellular domain.

The potential of EGFR-directed therapy to treat patients with HNSCC has been validated in recent trials in which patients receiving cetuximab and radiation demonstrated improved survival and locoregional control, as opposed to treatment with radiation alone [16]. Similar improvements were observed with the addition of cetuximab to platinum based therapy in the EXTREME trial [18]. However, the increases in survival and tumor control resulting from the addition of cetuximab in these trials are still modest, often measured in months or weeks. For this reason identification of predictive markers for improved patient selection as well as development of more efficacious agents targeting this important pathway are necessary to achieve improved outcomes in HNSCC patients.

One reason response to EGFR-directed therapy may be low is the cooperation and signaling redundancy between different members of the ErbB receptor family [19]. Despite the inhibition of even the most highly expressed family member (as is the case when cetuximab inhibits EGFR), proliferation may remain unimpeded because alternative signaling from other receptors are maintaining the activation the common downstream pathways shared by ErbB receptor family members. Thus, targeting multiple members of the ErbB receptor group is a rational approach, especially in subjects whose disease has initially progressed or have developed resistance to cetuximab therapy. This notion is support by breast cancer patients who experienced tumor progression after treatment with trastuzumab (Herceptin, Genentech), a monoclonal antibody targeted against HER2, have demonstrated responses to the dual EGFR and HER2 tyrosine kinase inhibitor lapatinib (Tykerb, GSK) [20].

Dacomitinib (Pfizer) is a second generation Pan-ErbB inhibitor that irreversibly binds several members of the HER family, including ErbB-1, ErbB-2 and ErbB-4 [21]. Significant in-vitro responses were observed with low concentrations of dacomitinib in lung cancer cell lines resistant to gefitinib [22] and in breast cancer cell lines resistant to trastuzumab and lapatinib [23]. In the clinic, a phase I dose escalation study in patients with advanced malignant solid tumors demonstrated well tolerated doses with significant antitumor activity [24]. Recent Phase I and II trials in advanced NSCLC have shown promising clinical activity as measured by disease stabilization and improved progression-free survival in patients that progressed on platinum therapy and were previously treated with erlotinib [25]. With its improved pharmacokinetic properties, including increased bioavailability, half-life, and lower clearance as compared to first generation irreversible Pan-ErbB inhibitors such as CI-1033, dacomitinib is an attractive agent for potential clinical use in HNSCC [21]. The goal of the current study was to determine in-vitro anti-proliferation effects of Dacomitinib in HNSCC cell lines. This includes the elucidation of mechanisms that explain the activity ErbB directed therapy in HNSCC cell line models as well as benchmarking its effectiveness against the only FDA approved targeted therapy for HNSCC treatment.

## Results

### Cell Lines Resistant to Cetuximab are Responsive to Dacomitinib at Low Concentration

A panel of 27 HNSCC cell lines reflecting the anatomical heterogeneity of the disease was used to test the antiproliferative effects of dacomitinib (PF-00299804) and cetuximab, the only FDA approved targeted therapy in HNSCC.(Table 1).

**Table 1:** Panel of HNSCC cell lines showing growth-inhibition effects of dacomitinib and cetuximab, mutation status of K-RAS and PIK3CA hotspots (as detected by PCR and sequencing), EGFR amplification status as detected by FISH (presented as ratio of EGFR gene to centromere 7), and anatomical category of original tumor primary site.

Cell Line	Category	Dacom.IC50 g	SE	Cetux. %Inhib.	%SE	K-RAS	PIK3CA	EGFR*
UMSCC-8	Oral Cavity	0.001	N/A	111	4.2	WT	WT	N/A
HN5	Oral Cavity	0.003	N/A	101.3	12.2	WT	WT	N/A
SCC-9	Oral Cavity	0.007	0	97.7	5.6	WT	WT	N/A
CAL27	Oral Cavity	0.009	0	67.5	2.4	WT	WT	4;2**
FADU	Hypopharynx	0.045	0	56.4	1.8	WT	WT	4;4
SCC-25	Oral Cavity	0.054	0.01	58.4	5.9	WT	WT	N/A
UMSCC-25	Larynx	0.085	0.01	25.9	11.2	WT	WT	4;4
UMSCC-38	Oropharynx	0.097	0.04	41.2	4.8	WT	WT	6;5
UMSCC-22A	Hypopharynx	0.277	0.08	43.4	0.8	WT	WT	3;3
UMSCC-5	Larynx	0.309	0.17	43.7	0.9	WT	WT	6;2
UMSCC-47	Oral Cavity	0.404	0.03	30.7	6.1	WT	WT	N/A
UMSCC-4	Oropharynx	0.415	0.09	75.9	1.3	WT	WT	N/A
UMSCC-11A	Larynx	0.462	0.4	48.9	2.4	WT	WT	4;2
SCC-15	Oral Cavity	0.519	0.17	24.3	7.6	WT	WT	N/A
UMSCC-6	Oropharynx	0.57	0.07	28.2	12.6	WT	WT	4;3**
UMSCC-81A	Larynx	0.746	0.06	39	0.3	WT	WT	6;6
UMSCC-14A	Oral Cavity	0.852	0.34	26.2	2	WT	WT	5;5
UMSCC-12	Larynx	1.202	0.3	25.3	0.4	WT	WT	4;4
UMSCC-2	Oral Cavity	1.453	1.03	17.4	2	WT	WT	4;4
SCC-4	Oral Cavity	1.465	0.3	30.1	16.6	WT	WT	N/A
UMSCC-19	Oropharynx	1.567	0.33	28	3.9	WT	WT	N/A
UMSCC-11B	Larynx	1.731	0.19	32.4	2.2	WT	WT	6;7
UMSCC-7	Oral Cavity	1.733	0.2	25.6	1.6	WT	WT	4;4
UMSCC-1	Oral Cavity	1.871	0.1	8.7	1.2	WT	WT	5;5
UMSCC-17B	Larynx	2.14	0.32	0	0.8	WT	WT	3;3
UMSCC-74A	Oropharynx	2.547	0.04	5.8	0.4	G12D	WT	2;2
CAL33	Oral Cavity	3.097	0.21	47.2	5.1	WT	H1047R	8;4**

Categories encompass the following subsites: oral cavity; front 2/3 of tongue, floor of mouth, alveolar ridge. Hypopharynx; hypopharynx. Larynx; larynx, supraglottis. Oropharynx; base of tongue, tonsil, tonsillar pillar. IC50 g is Dacomitinib IC50 g and Cetux. % Inhib. is Cetuximab percent inhibition.

* EGFR:Centromere 7. Dacom.

** Ploidy and copy number were variable in these cell lines, and in the table we use the most common copy number.

**Figure 1:**
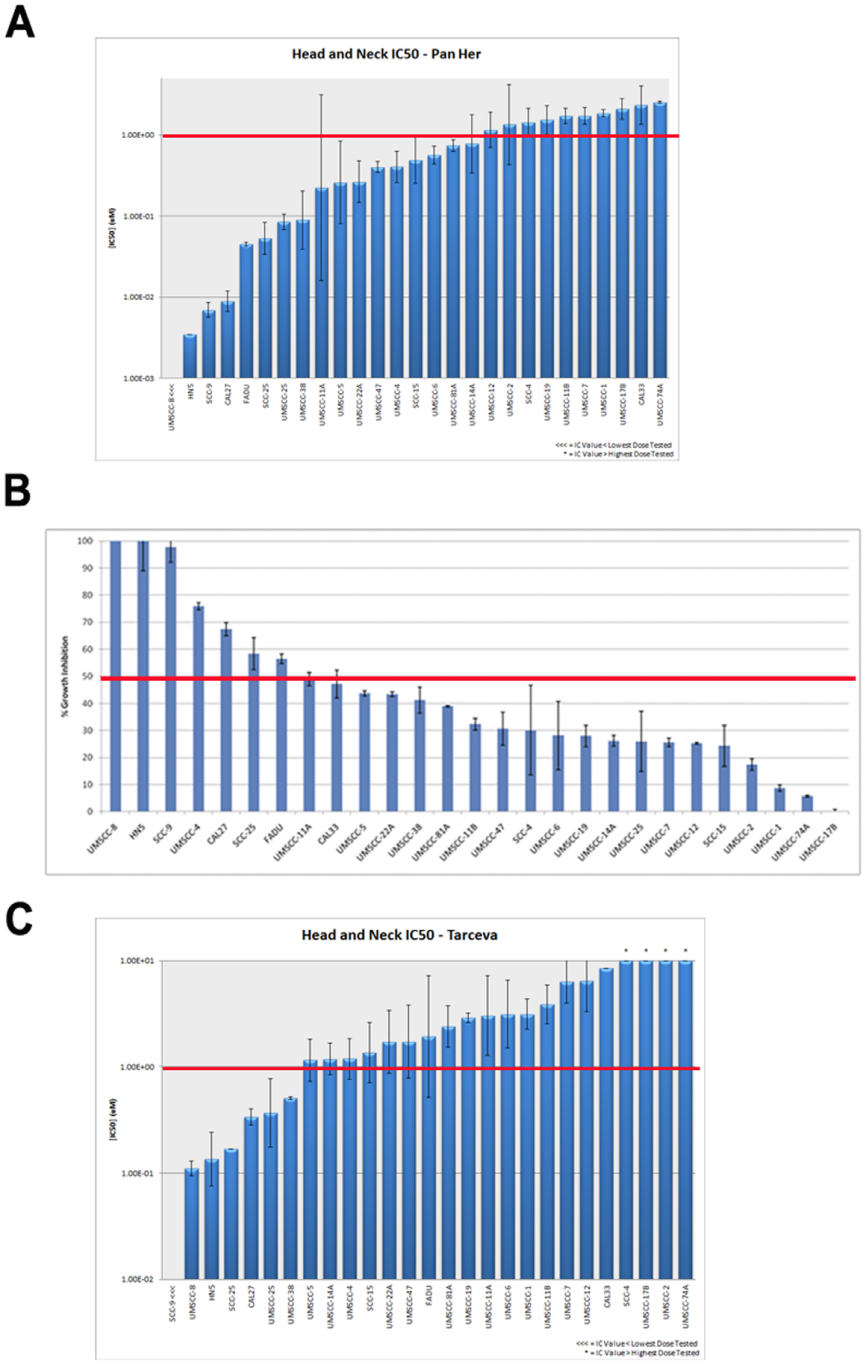
Growth-inhibitory effects of dacomitinib and cetuximab on head and neck cancer cell lines. Cells were counted after five days of treatment. SE bars were derived from experiments repeated at least twice. A. Dacomitinib IC50 g values are arranged from lowest to highest IC50 g on a log scale. Cells were treated at concentrations from 0.001 to 10 uM. B. Percentage growth inhibition with cetuximab treatment. Cells were treated at a fixed dose of 100 ug/mL. C. Erlotinib IC50 g values are arranged from lowest to highest IC50 g on a log scale. Cells were treated at concentrations from 0.015 to 10 uM. Red indicates the sensitivity cutoff. For dacomitinib and erlotinib, the sensitivity cutoff is set at 1 uM, and for cetuximab it’s set at 50%.

Dacomitinib inhibited the growth of all head and neck cancer cell lines in a concentration-dependent manner. However, there was significant heterogeneity in IC50 g values across the panel, with a 4 log-fold difference between the most sensitive and least sensitive cell lines (Figure 1a). A cutoff of 1 uM was used to stratify responsive cell lines based on a previous study in the breast cancer cell line panel [23]. 17/27 cell lines were defined as dacomitinib responsive and had an IC50 g less than 1 uM. Treatment with 100 ug/ml of cetuximab resulted in greater than 50% inhibition in 7/27 cell lines (Figure 1b). The response of cell lines to either compound did not correlate with the primary tumor anatomical site.

In addition, the same panel used to assess the sensitivity of cetuximab and dacomitinib in HNSCC cells was used to assess the sensitivity of erlotinib, an EGFR specific small molecule tyrosine kinase inhibitor. Although erlotinib is not an FDA approved therapy in HNSCC, it is in a similar class of targeted therapy as dacomitinib. Thus to assess if the difference between between dacomitinib and cetuximab observed above is related to the pharmacological differences between the drug classes, ie antibody versus small molecule inhibitor, rather than their biological targets we also assessed the sensitivity of erlotinib. Only 25.6% (7 out of 27) HNSCC cell lines were erlotinib responsive and had an IC50 less than 1 uM (Figure 1C). This rate of highly sensitive cell lines (defined as those with IC50 less than 1 uM) was (the term “response rate” should be reserved for an actual clinical study utilizing the usual complete/partial total to generate that value. It is specific for this and we shouldn’t confuse the readers in a preclinical study unless it is of tumor xenograft study etc).

Similar to that of cetuximab such that only 7 out of 27 HNSCC cell lines also had greater than 50% inhibition with 100 ug/ml of cetuximab treatment. This is in stark contrast to dacomitinib which achieved a highly sensitive rate of 62.9% with the same 1 uM sensitivity cutoff. Of the seven HNSCC lines sensitive to erlotinib (IC50<1 uM), five had greater than 50% inhibition after treatment with 100 ug/ml of cetuximab. The other two cell lines (UMSCC-25 and UMSCC-38) which had an IC50 g of 0.367 and 0.508 uM respectively had 25.9% and 41.2% inhibition with 100 ug/ml of cetuximab treatment. There were two cell lines which had greater than 50% inhibition after treatment with cetuximab but did not have less 1 uM IC50 g with erlotinib. The cell lines UMSCC-4 and FADU which had an 75.6% and 56.4% inhibition with cetuximab treatment, had IC50 g of 1.19 and 1.192 uM respectively after treatment with erlotinib. All seven of the erlotinib responsive cell lines were also dacomitinib responsive. Of the remaining 20 cell lines, 16 had IC50 g between 1 uM and 10 uM and 4 cell lines did not achieve IC50 g at the maximum tested concentration of 10 uM.

HNSCC sensitivity to erlotinib was similar to cetuximab sensitivity such that only 25.6%(7/27) of the panel were responsive to erlontinib and cetuximab whereas 62.9%(17/27 HNSCC had IC50 g<1 uM) was responsive to dacomitinib. Since cetuximab has demonstrated clinical value and is thus a standard of care for head and neck cancer, the rest of the comparison was done between dacomitinib and cetuximab.

### Common Oncogene Mutations and Amplifications are Rare in HNSCC and do not Correlate with Sensitivity to Cetuximab nor Dacomitinib

Mutations in EGFR, K-Ras and PI3K have been shown to affect response to targeted therapies [4], [26]. However, these mutations are not abundant in head and neck tumor samples [27], [28]. We wanted to assess if 1) our cell line panel was reflective of the same trend and 2) if mutations in these genes were associated with sensitivity to cetuximab and dacomitinib. We performed mutation analysis on the panel of head and neck cancer cell lines used in the proliferation analysis and found that mutations were rare and could not be used to explain sensitivity to cetuximab and dacomitinib (Table 1). None of the cell lines harbored EGFR mutations in exons 19 nor 21. Only the CAL-33 cell line exhibited a mutation in exons 9 or 20 of PI3K (a heterozygous H1047R mutant) and only UMSCC-74A exhibited a KRAS mutation (a heterozygous G12D mutant). Both of these cell lines had a lower sensitivity to dacomitinib although CAL33 was somewhat responsive to cetuximab.

Amplification of receptor tyrosine kinases has been associated with sensitivity to therapies targeting these receptors [29]. Previous studies have found up to 20% of HNSCC are EGFR amplified, but the prognostic and predictive value of this aberration has been mixed [30]. We performed FISH analysis to assess the copy number of EGFR in 19 out of the 27 cell lines. None of the cell lines were amplified for EGFR and only 4/19 cell lines (UMSCC-5, UMSCC-11A, CAL-27 and CAL-33) showed increased copy number of EGFR (Table 1). Interestingly, increased copy number of EGFR was not associated with sensitivity to dacomitinib; one of the most sensitivity lines (CAL-27) and one of the most resistant lines (CAL-33) were among the four lines with gains of EGFR.

### Baseline Phosphorylated and Total EGFR Levels with and without EGF Atimulation Correlated with Sensitivity to EGFR-directed Therapy

To assess the degree to which dacomitinib and cetuximab block EGFR signaling in head and neck cancer cell lines and ascertain if its effectiveness in blocking EGFR is associated with their ability to inhibit growth, we performed Western blot analysis to assess protein levels and phosphorylation status of key molecules in the EGFR signaling pathway.

**Figure 2:**
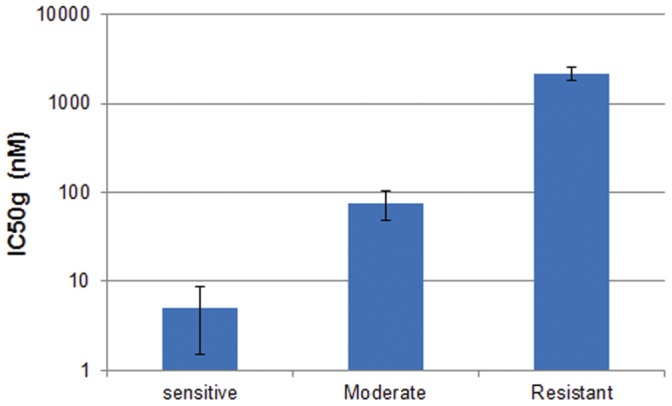
Dacomitinib IC50 g of ten head and neck cancer cell lines representing three different levels of sensitivity to dacomitinib on a log scale. Four cell lines were selected from the exquisitely sensitive group (IC50 g <10 nM), three from the moderate group (IC50 g10 nM - 1 uM) and three from the resistant group (IC50 g >1 uM). These ten lines are used in the Western blot and flow cytometry experiments. Average IC50 g of the selected sensitive lines is 5 nM; moderate 75 nM; resistant 2186 nM.

**Figure 3:**
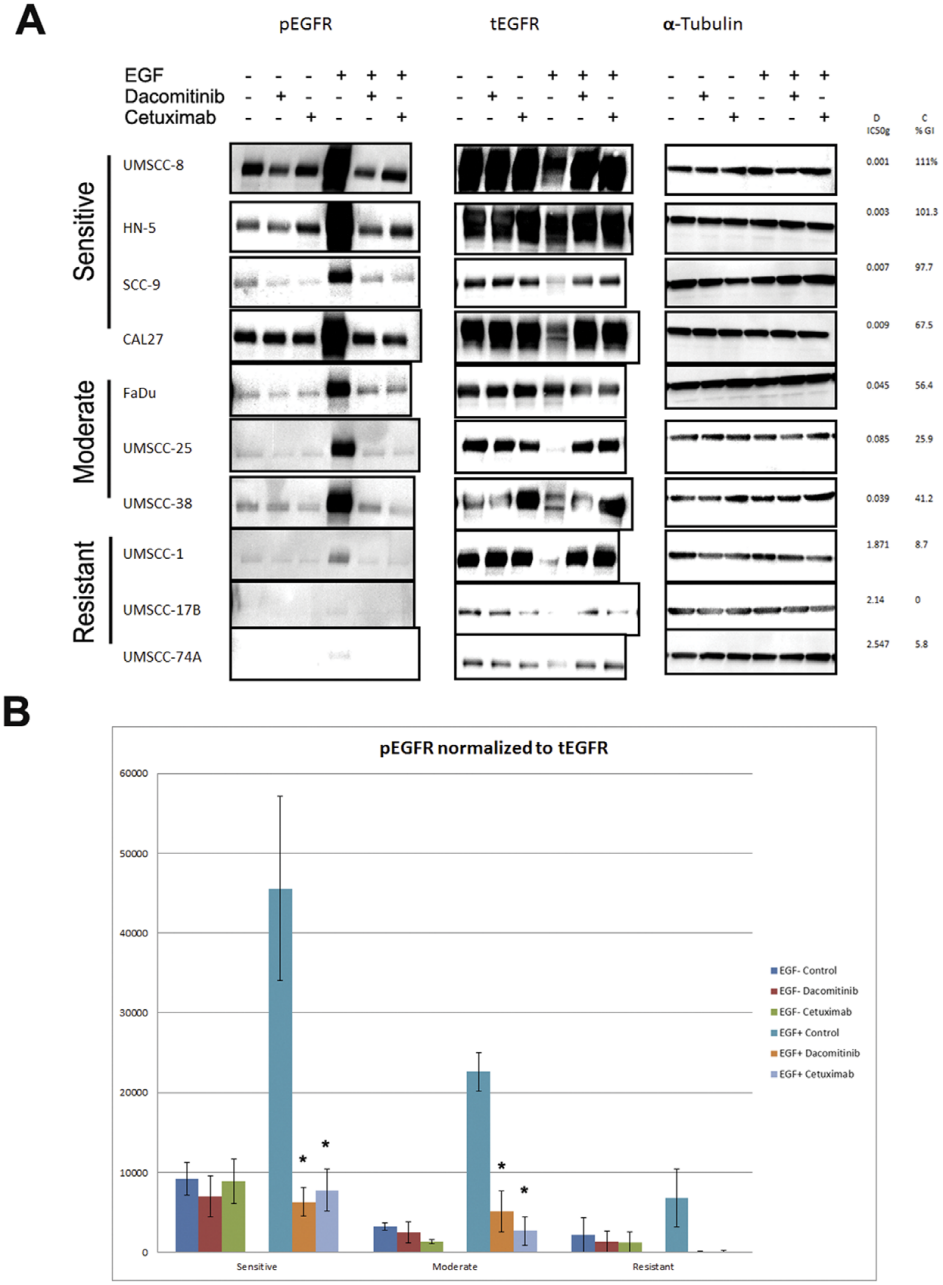
A. Effects of dacomitinib and cetuximab on phosphorylated and total EGFR. Cells were cultured to log-phase and treated with 100 nM dacomitinib or 100 ug/mL cetuximab for 1 hour, with or without treatment with 10 ng/mL recombinant EGF ligand. Cells lysates were then harvested, and protein was resolved using Western blot analysis. B. Western blot images were quantified using ImageJ software. Protein levels were quantitated for each cell line and were averaged by group. Phosphorylated EGFR was normalized to total EGFR. *. p<0.05, student’s t-test.

We performed Western blot analysis on a subset of cell lines representative of the differential response to dacomitinib treatment. Four lines were selected from the most sensitive group (IC50<10 nM), three lines from the moderate group (1 uM>IC50>10 nM) and three lines from the resistant group (IC50>1 uM). The average dacomitinib IC50 g of the selected cell lines in each group is displayed in Figure 2. Cells were treated with either 100 nM dacomitinib or 100 ug/mL cetuximab in both EGF-stimulated and unstimulated states. Both total and phosphorylated EGFR (tEGFR and pEGFR) protein levels were assessed (Figure 3a). Total EGFR level was associated with sensitivity to dacomitinib; three of the four lines in the highly sensitive group had the highest levels of phosphorylated and total EGFR among the ten lines selected, while the three resistant lines collectively had the lowest levels (Figure 3a). Cell lines in the moderate group had intermediate levels of phosphorylated and total EGFR. To compare pEGFR levels between the sensitive, moderate and resistant groups, Western blot images were quantified and the average level of pEGR for each sensitivity group were calculated and normalize. Treatment with either compound significantly blocked levels of EGF-stimulated pEGFR in the highly sensitive and moderate groups, but not in the resistant group (Figure 3b).

Treatment with either drug did not reduce total EGFR levels (Figure 3a). However, there was a notable reduction in total EGFR in the control cells with EGF stimulation. It is known that EGFR is internalized and often degraded after stimulation by EGF or other ligands, which may explain our observation [31]. Addition of either drug, however, negated this effect, which adds evidence that compounds which bind EGFR may inhibit internalization and degradation.

### Dacomitinib but not Cetuximab Inhibits EGF Stimulated EGFR Downstream Pathways

**Figure 4:**
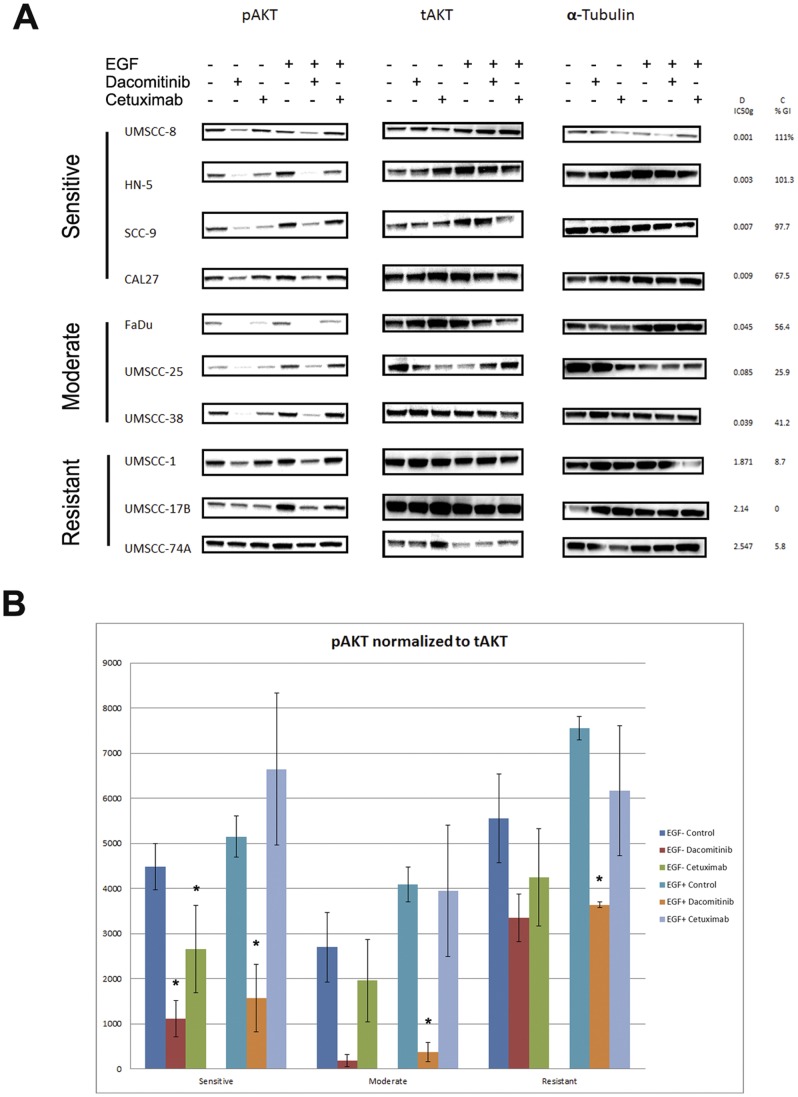
A. Effects of dacomitinib and cetuximab on phosphorylated and total AKT. Cells were cultured to log-phase and treated with 100 nM dacomitinib or 100 ug/mL cetuximab for 1 hour, with or without treatment with 10 ng/mL recombinant EGF ligand. Cells lysates were then harvested and protein was resolved using Western blot analysis. B. Western blot images were quantified using ImageJ software. Protein levels were quantitated for each cell line and were averaged by group. Phosphorylated AKT was normalized to total AKT. *. p<0.05, student’s t-test.

The PI3K-AKT-mTOR and Ras-Raf-MAPK signaling pathways are downstream effectors of EGFR signaling [8]. We wanted to evaluate the effect of dacomitinib and cetuximab on these pathways. First, we assessed the protein levels of total AKT (tAKT) and phosphorylated AKT (pAKT) upon treatment with either dacomitinib or cetuximab. In control conditions, neither pAKT nor tAKT levels were associated with sensitivity (Figure 4A) in either baseline or EGF stimulated condition. EGF stimulation significantly increased pAKT in all cell lines.

Dacomitinib reduced pAKT levels in all seven cell lines in the sensitive and moderate groups and one cell line in the resistant group (UMSCC-1) in both baseline and EGF stimulated conditions (Figure 4A). Dacomitinib was able to reduce pAKT levels in resistant cell line UMSCC-17B in the EGF stimulated conditions only. In the resistant cell line (UMSCC-74A) pAKT levels were slightly reduced in the EGF stimulated condition. Cetuximab treatment was less potent at reducing pAKT. Treatment with cetuximab caused reduction in pAKT in only five out of ten cell lines (2/4 sensitive lines, 3/3 moderate lines and 0/3 resistant lines), and the level of reduction was significantly less than those caused by treatment with dacomitinib, regardless of EGF stimulation (Figure 4a).

As with EGFR, Western blots were quantified and group specific levels of pAKT normalize to tAKT were determined and compared (Figure 4b). Without EGF stimulation, both compounds significantly reduced pAKT levels in the highly sensitive group and not the moderate or resistant groups. In EGF stimulated conditions, dacomitinib was able to reduce pAKT levels in all three groups. In all tested scenarios, dacomitinib caused greater reductions in pAKT levels than cetuximab.

**Figure 5:**
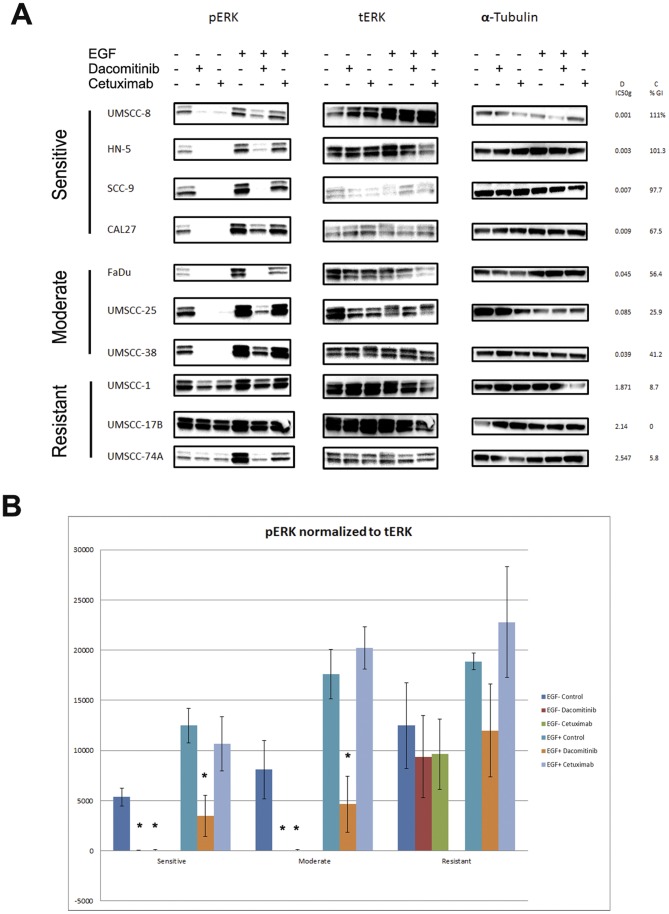
A. Effects of dacomitinib and cetuximab on phosphorylated and total ERK. Cells were cultured to log-phase and treated with 100 nM dacomitinib or 100 ug/mL cetuximab for 1 hour, with or without treatment with 10 ng/mL recombinant EGF ligand. Cells lysates were then harvested and protein was resolved using Western blot analysis. B. Western blot images were quantified using ImageJ software. Protein levels were quantitated for each cell line and were averaged by group. Phosphorylated ERK was normalized to total ERK. *. p<0.05, **. p<0.01, student’s t-test.

Activation of Ras by EGFR signaling initiates a multistep phosphorylation cascade that leads to the activation of MAPKs, ERK1 and ERK2, and ultimately regulates transcription of genes involved in cell proliferation. Total and phosphorylated ERK (ERK1 and 2) protein (tERK and pERK)levels were analyzed upon treatment with either dacomitinib or cetuximab. In the control conditions, tERK and pERK protein levels were lower in the highly sensitive group and higher in the resistant group (Figure 5a and 5b) regardless of EGF stimulation. Treatment with either compound significantly reduced levels of pERK in the seven cell lines in the highly sensitive and moderate groups, and only 1(UMSCC-1) out of three cell lines in the resistant group in the baseline condition (Figure 5a). Upon stimulation with EGF, pERK levels significantly increased across all cell lines. Under this condition, Dacomitinib still blocked phosphorylation of ERK levels in all seven cell lines in the sensitive and moderate group. In addition to reducing pERK levels in the resistant line UMSCC-1, under EGF stimulated condition, dacomitinib also reduced pERK levels in the resistant line UMSCC-74A. Cetuximab was only able to reduce pERK levels in one sensitive (SCC-9), one moderate (FaDu) and one resistant (UMSCC-74A) line. In all scenarios, dacomitinib caused greater reductions in pERK levels than cetuximab.

Western blots were quantified and group specific levels of pERK normalize to tERK were determined and compared (Figure 5b). Without EGF stimulation, both compounds significantly reduced pERK levels in the highly sensitive group and moderate groups but not resistant group. In EGF stimulated conditions, only dacomitinib was able to reduce pERK levels. In all tested scenarios, dacomitinib caused greater reductions in pERK levels than cetuximab.

### Dacomitinib Causes Greater G0/G1 Arrest than Cetuximab

Dacomitinib and cetuximab may have antiproliferative activity by inducing cell cycle arrest and apoptosis. The same ten cell lines selected for the Western blot assays, representing the varying proliferation responses to the two compounds, were used to analyze effects on the cell cycle and apoptosis.

**Figure 6:**
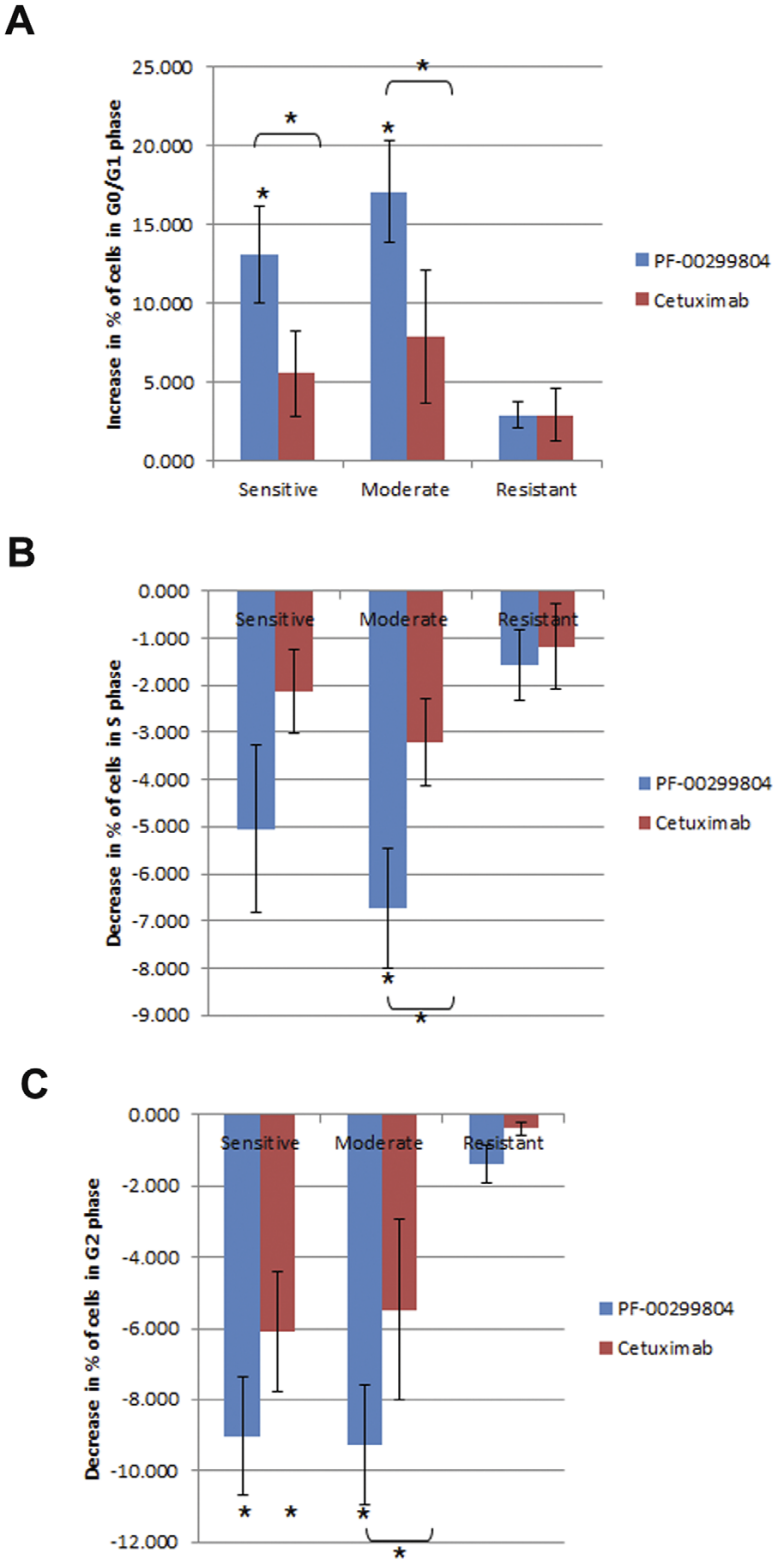
Effects of dacomitinib and cetuximab on cell cycle. Cells were treated with 100 nM dacomitinib or 100 ug/mL cetuximab for five days before analysis using flow cytometry. Data shown is average percentage of cells in the group assignments from Figure 2. A. Change in percentage of cells in G0/G1 phase. B. Change in percentage of cells in S phase. C. Change in percentage of cells in G2 phase. *. p<0.05, student’s t-test.

Dacomitinib caused greater cell cycle arrest than cetuximab. After treatment with 100 nM of dacomitinib or 100 ug/mL cetuximab for five days, there was a significant increase in percentage of cells in G0/G1 phase in the sensitive and moderate groups with dacomitinib but not with cetuximab as compared to control (Figure 6a). Although cetuximab did cause some G0/G1 arrest, this effect was not significant compared to control and significantly lower than the effect of dacomitinib in both the sensitive and moderate groups. There was less than five percent decrease in S phase in all the conditions except for the dacomitinib treated moderate group which had a significant decrease (Figure 6b). The general trend of the effects on S phase mimicked the effects on G0/G1 phase. Consistently, dacomitinib treatment of the sensitive and moderate groups caused a greater decrease in G2 than cetuximab treatment (Figure 6c).

**Figure 7:**
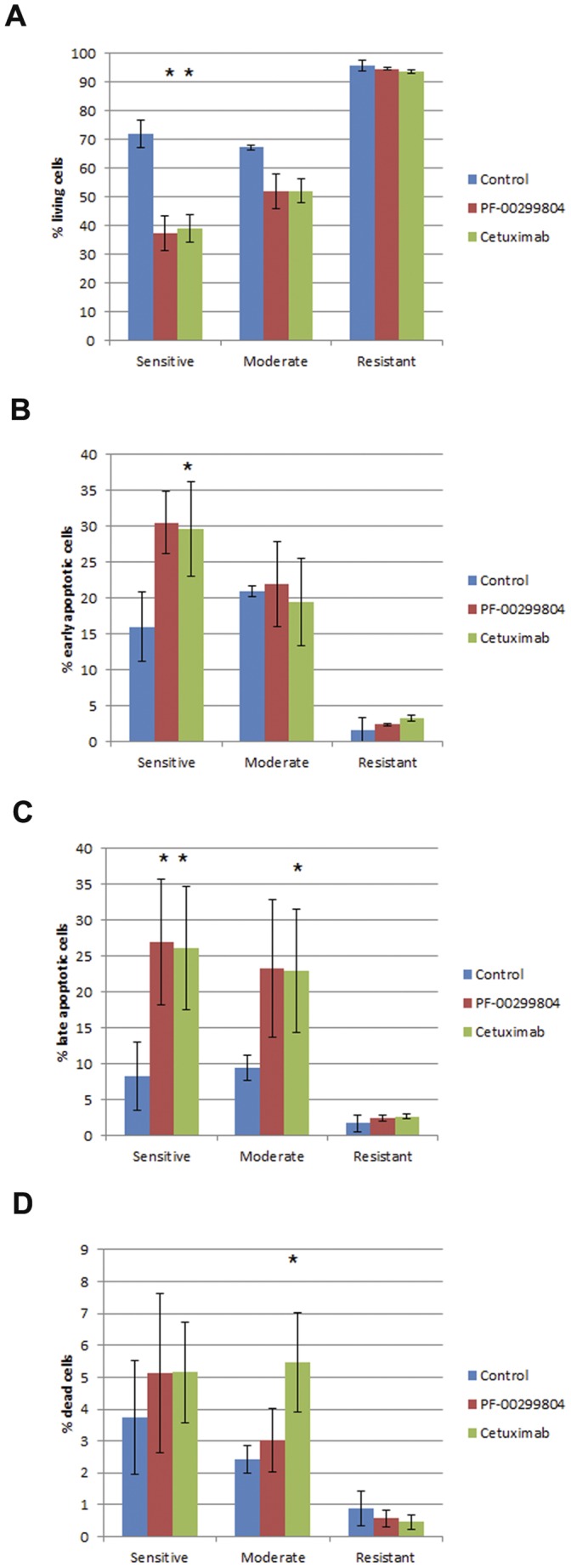
Dacomitinib and cetuximab effect on apoptosis. A. Changes in % living cells. B. Changes in cell in early apoptosis. C. Changes in cells in late apoptosis. D. Changes in % dead cells.

Dacomitinib was as effective as cetuximab at inducing apoptosis. In the sensitive and moderate groups, both compounds caused a decrease in percentage of cells that were living (Figure 7A). There was a concomitant increase in percentage of cells that were in an early or late apoptotic state (Figure 7B, Figure 7C). Less than 6% of cells were dead in any of the experiments (Figure 7D). Neither drug caused significant apoptosis in the resistant group. There was not a significant difference between the compounds in inducing apoptosis.

### Dacomitinib is More Effective at Blocking EGFR Signaling than the Small Molecule Tyrosine Kinase Inhibitor Erlotinib

As stated earlier, response to highly specific EGFR-directed therapy may be low because there maybe cooperation and signaling redundancy between different members of the ErbB receptor family. Thus, we assessed dacomitinib, a panHER inhibitor with broad specificity, in a panel of 27 HNSCC and showed HNSCC cell lines are more sensitive to it than the small molecule anti-EGFR tyrosine kinase inhibitor erlotinib and the anti-EGFR antibody cetuximab in proliferation assays (see Figure 1) and using a subset of ten cell lines we showed that dacomitinib was more effective at blocking EGFR signaling than cetuximab. The characterization of dacomitinib was done in comparison with cetuximab, the only FDA approved anti-EGFR agent in the treatment of HNSCC. However, erlotinib and dacomitinib are both small molecule inhibitors as opposed cetuximab which is an antibody and thus erlotinib’s ability to block EGFR signaling was assessed using Western blotting.

**Figure 8:**
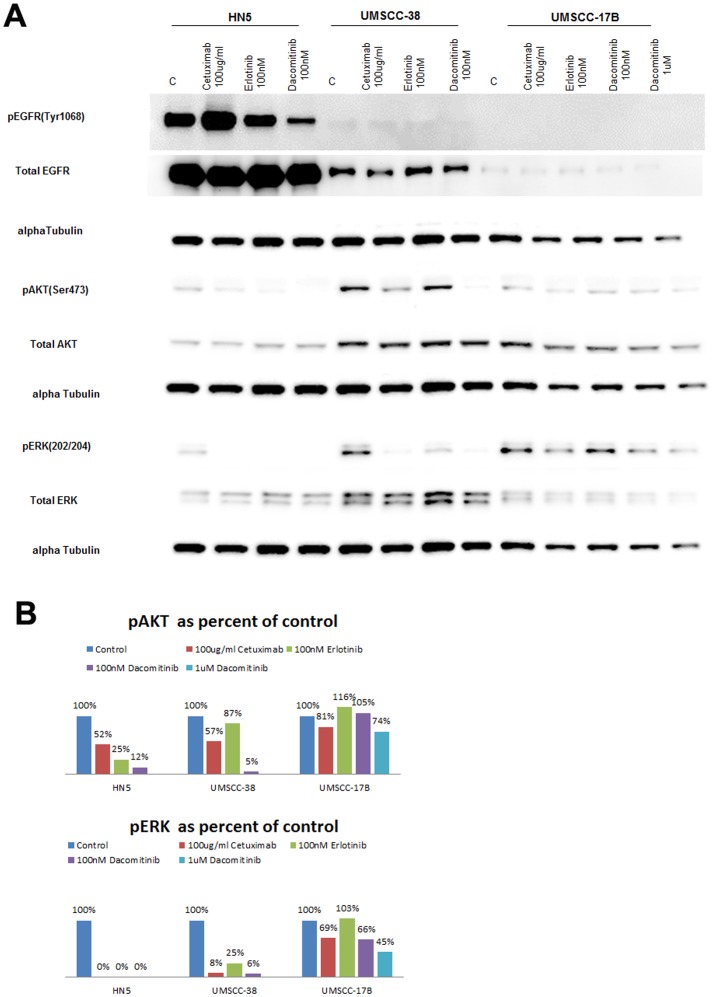
A. Effects of dacomitinib, erlotinib and cetuximab on phosphorylated and total EGFR, ERK, and AKT. Cells were cultured to log-phase and treated with 100 nM dacomitinib, 100 nM erlotinib or 100 ug/mL cetuximab for one hour. Cells lysates were then harvested and protein was resolved using Western blot analysis. B. Western blot images were quantified using ImageJ software. Protein levels were quantitated for each cell line. Phosphorylated ERK and AKT were normalized to alpha tubulin and presented as a % of the control.

Three cell lines representing the three classes of sensitivity described before were examined. In the sensitive line HN5 dacomitinib was more effective at blocking EGFR signaling than either cetuximab or erlotinib (Figure 8A). Only HN5 cells treated with 100 nM of dacomitinib had significantly lower levels or pEGFR compared to control. EGFR signaling in the moderately sensitive line, UMSCC-38 and UMSCC-17B was low.

More significant changes were noticed in the downstreams PI3K-AKT-mTOR and Ras-Raf-MAPK pathway, as dacomitinib was significantly more effective at blocking both phosphorylation of AKT and ERK (Figure 8A). Western blots were quantified and pAKT and pERK levels were presented as percent of control after normalizing to alpha tubulin to control for total protein (Figure 8B). HN5 cell treated with 100 nM of erlotinib had twice as much pAKT as those treated with 100 nM of dacomitinib. UMSCC-38 cell treated with 100 nM of erlotinib had 17 times more pAKT than those treated with the same concentration of dacomitinib. Similarly, although all three drugs were effective a blocking phospho ERK in the sensitive line HN5, UMSCC-38 treated with 100 nM of erlotinib had 4 times more pERK signal than UMSCC-38 cells treated with the same concentration of dacomitinib. Erlotinib was less effective at blocking both phospho ERK and AKT in the UMSCC-38 compared to cetuximab and dacomitinib. In the resistant line UMSCC-17B, neither erlotinib nor dacomitinib had significant effect on pAKT but dacomitinib was more effective at blocking pERK level than erlotinib (Figure 8B).

To assess if a dose dependence might be seen using a higher concentration of dacomitinib (1 uM) was tested at blocking ERK and AKT mediated signaling in the resistant line, UMSCC-17B. Compared to 100 nM treatment, UMSCC-17B cells treated with 1 uM dacomitinib had a 25% reduction in pAKT and 21% reduction in pERK (Figure 8A and 8B).

## Discussion

The irreversible pan-ErbB inhibitor dacomitinib is currently in Phase III clinical trials for the treatment of patients with lung cancer and has shown promising activity in treating this malignancy (http://clinicaltrials.gov/ct2/show/NCT01000025). We sought to compare the effects of dacomitinib with cetuximab, an EGFR inhibitor that is the currently the only FDA-approved targeted therapy for HNSCC treatment and to erlotinib a small molecule EGFR inhibitor not yet FDA approved in HNSCC. We provide evidence that treatment with a pan-HER inhibitor such dacomitinib is more effective than using the EGFR specific inhibitors cetuximab and erlotinib.

Our data demonstrate the effectiveness of dacomitinib in abrogating growth of HNSCC cell lines in vitro. Our data shows that cell lines exhibiting dramatic growth inhibition with cetuximab also exhibited significant growth inhibition with dacomitinib, which demonstrates, at the very least, the lack of inferiority of dacomitinib compared to cetuximab. Of clinical interest is that 3/27 cell lines, 11% of the panel, experienced greater than 90% growth inhibition with cetuximab, which is reminiscent of the clinical observation of a 10% response rate observed in patients receiving cetuximab therapy. These three cell lines were exquisitely sensitive to dacomitinib as well with IC50 g values less than 10 nM. If this preclinical observation holds clinically, we would predict those patients that respond to cetuximab would also respond to dacomitinib. Testing the oral dacomitinib in patients with HNSCC would complement the already commercially available intravenous antibody cetuximab as it offers the potential as an alternative or possibly complementary treatment. An oral agent would obviate the need for patients to receive frequent intravenous therapy, the need for suitable facilities at which to administer treatment, inherent economic resources as well as serious side-effects inherent in intravenous antibody treatment such as infusion reactions. One next step would be to assess the effectiveness of dacomitinib in animal models of HNSCC.

Our data also showed that improvement in responsive to EGFR inhibition with a Pan Her TKI (dacomitinib) is not simply related to the fact that dacomitinib is a small molecule inhibitor whereas cetuximab is an antibody. There are real pharmacologic differences between small molecule inhibitors and antibodies. Antibodies (∼150 kDa) are much larger than small molecule inhibitors (50 kDa). For example, in brain cancer, antibodies are ineffective at crossing the blood brain barrier and thus to utilize such therapy in patients with brain cancer, techniques such as intrathecal or intra-tumoral administration must be considered [32]. Small molecule inhibitors are administered orally and tend to have a much shorter half-life than antibodies [33]. More significantly, antibodies and small molecule inhibitors often target different parts of a protein. Cetuximab binds to the ligand-binding domain in the ectodomain of EGFR whereas the small molecule inhibitors gefitinib and erlotinib specifically inhibit EGFR phosphorylation by functioning as ATP analogues and competing with ATP binding within the catalytic domain [34], [35], [36]. Even though we tested an EGFR specific small molecule TKI (erlotinib), the number of responsive cell lines did not increase. There were still only 25% of the panel that were considered responsive to EGFR inhibition. Furthermore, all of the cell lines tested with both erlotinib and dacomitinib showed greater sensitivity to dacomitinib which is further evidence that a pan her inhibitor maybe an improvement over using EGFR specific treatment. Further in vivo comparisons needs to be performed to assess toxicity and specificity.

The goal of the project was to generate pre-clinical rationale for the development of dacomitinib in head and neck cancer. To that end the current, and only approved EGFR agent, cetuximab, was selected for comparison. Previously, another study had compared lapatinib, a small molecule tyrosine kinase inhibitor, to trastuzumab, an FDA antibody [37] used to treat breast cancer. This preclinical study provided support to the development of lapatinib as an approved agent in breast cancer. Recently, our laboratory group provided preclinical rational for using dacomitinib in HER2 amplified breast cancer cell lines resistant to trastuzumab and lapatinib [23].

A sensitivity cutoff of 1 uM was used to distinguish sensitive HNSCC cell line. This cutoff is similar to the cutoff used to distiniguish sensitive cell line in the breast cell line panel [23]. It has been observed that 1 uM is where off-targeting or non-specific effects begin to manifest based on the enzymatic analysis of the drug. Anti-proliferative effects below 1 uM are therefore more likely to be due to the specific effect of the drug on its designed molecular target. Furthermore, PK data from a Phase I study [24] demonstrated that the maximum plasma levels of dacomitinib were between 200–300 nM which is within the range of our sensitivity cutoff. However, it is noteworthy to mention that there are many factors that make it difficult to generalize in vitro drug concentration into the clinical setting. Therefore, any cut-off for in vitro sensitivity is going to be somewhat arbitrary.

Several of the HNSCC cell lines in which cetuximab inhibited growth by less than 50% had dacomitinib IC50 g values of less than 100 nM. In a recent phase I study, the maximum plasma levels of dacomitinib was between 200 nM-300 nM and thus using 100 nM of dacomitinib for the biochemical analysis in our current study is within that range, erring on the conservative side [24]. We also demonstrated that in the presence of an EGFR ligand, cetuximab does not inhibit pathways involved in cell growth, whereas dacomitinib significantly inhibits these pathways. In the clinic, high levels of EGFR ligands such as TGF in HNSCC patients have been associated with worse patient outcomes [13]. Thus this compound may have potential for the treatment in patients with HNSCC especially those who initially progressed despite cetuximab therapy or ultimately developed resistance after initial response.

Resistance to dacomitinib and cetuximab does not appear to be mediated by ligand independent signaling. EGFRviii is a truncated form of EGFR which is associated with tumorgenicity and resistance to treatment. Since EGFRviii is constitutively active regardless of the presence of ligand [38], [39], it might be postulated that cells with EGFRviii mediated resistance to have higher levels of basal phosphorylated EGFR than those that are sensitive. In our cell lines we observe the converse; cells sensitive to dacomitinib have significantly higher levels of basal phosphorylation than resistant cell lines. Furthermore, one might predict the increase in baseline phosphorylation of EGFR to be less responsive to EGF stimulation in the resistant cell lines compared to the sensitive cell lines. We did not observe this trend either. The addition of the EGFR ligand, EGF, increased phosphorylation of EGFR in both the sensitive and resistant cell lines. These observation indicate that the ligand independent EGFRviii signaling may not be associated with resistant to EGFR therapy although one has to take into consideration conformational differences of a truncated receptor when developing ad testing inhibitors.

Our cell line panel generated from human head and neck cancer tumor specimens not surprisingly exhibits a similar biomarker profile that is seen in HNSCC biopsies and tumor specimens. The lack of exon 19 and 21 EGFR mutations and low frequency of K-RAS and PI3K mutations present in our panel in consistent with the molecular characteristics observed in HNSCC patients [27], [28]. This provides support that our panel is a suitable study model to perform preclinical studies in head and neck cancer. The two cell lines possessing either mutation were among the least sensitive cell lines to dacomitinib. These activating mutations of signaling molecules downstream of EGFR suggests that inhibition of downstream effectors of the mutated component may be required to abrogate growth in these cell lines. This strategy is currently being pursued in a clinical trial investigating the efficacy of a MEK inhibitor in patients harboring activating mutations in the RAF oncogene, a gene which like K-RAS is a downstream effector of EGFR signaling. (http://clinicaltrials.gov/ct2/show/NCT00888134). The predictive value of such mutations in HNSCC remains limited, however, as no clinical studies have investigated outcomes in HNSCC patients with or without mutations receiving EGFR directed therapy. In other histologies, the predictive value of such mutations has been inconsistent. K-RAS mutations in colorectal cancers have been clinically shown to render tumors insensitive to the EGFR-directed antibodies cetuximab and panitumumab, whereas the EGFR-directed small molecule erlotinib has obtained FDA approval for the treatment of pancreatic cancer, of which over 70–90% possess K-RAS mutations [4], [40]. Further studies of inhibitors targeting mutated effectors of EGFR and molecules further downstream may elucidate mechanisms by which HNSCC cells possess or acquire resistance to EGFR directed therapy.

### Conclusion

The goal of this preclinical investigation was to study the effects of dacomitinib on the growth of HNSCC cells and to compare this compound with cetuximab, the currently utilized molecular therapy in the clinical treatment of HNSCC. Analyzing the data from the current study as well as the promising activity of the investigational compound in current trials, there is strong evidence to consider evaluating dacomitinib for the treatment of patients with HNSCC.

Furthermore, data from this study provides evidence that our head and neck cell line panel is a reasonable study model to perform preclinical studies in head and neck cancer. It is our hope that these preclinical data will provide a foundation of information which will translate toward meaningful clinical value such improved responses to EGF directed therapy and better management of HNSCC.

## Materials and Methods

### Cell Lines, Cell Culture and Reagents

The effects of dacomitinib, erlotinib and cetuximab on growth were studied in 27 HNSCC cell lines in vitro. Lines prefixed with the UMSCC- designation were obtained from the University of Michigan (Ann Arbor, MI, USA) [41]. CAL27, CAL33, FaDu, SCC-4, SCC-9, SCC-15 and SCC-25 were obtained from ATCC (American Type Culture Collection, Rockville, MD, USA). HN5 was a kind gift from OSI Pharmaceuticals (Melville, NY, USA). Cells were cultured in D-MEM media (ATCC) supplemented with 10% heat-inactivated fetal bovine serum, 2 mmol/L glutamine and 1% antibiotic-antimycotic solution (Gibco/Invitrogen, Carlsbad, CA, USA). Before any experiments were performed, all cell lines were screened for mycoplasma using previously established methods [42]. Mitochondrial DNA regions of each cell line were also sequenced to confirm individuality using previously established methods [43].

### Proliferation Assays

Cells were seeded in duplicate in 24-well plates at densities of 10,000 to 25,000 cells per well. Cells were treated 24 hours after initial seeding. Dacomitinib(gift from Pfizer) and erlotinib (aka Tarceva –gift from Genentech) was added at 10 uM with two fold dilutions over nine dilutions (ranging from 10 uM to 0.039 uM) and cetuximab (commercially available) was added at a concentration of 100 ug/ml. At the time of treatment, one set of untreated cells was harvested via trypsinization and placed in isotone solution for immediate counting using a Coulter Z1 particle counter (Beckman Coulter Inc., Fullerton, CA, USA). The remaining wells were counted 6 days after seeding. Growth inhibition was calculated by percent generational inhibition [44]. All growth inhibition experiments were performed at least twice.

### EGFR, K-Ras and PI3K Mutation Analysis

Aliquots of each cell line were collected from culture, washed in PBS and then pelleted. Genomic DNA was extracted and purified using the DNeasy Blood & Tissue Kit (Qiagen, Germantown, MD, USA). PCR for exons 19 and 21 of EGFR, exon 1 of KRAS and exons 9 and 20 of PIK3CA were performed according to previously established methods [27]6. Primers were synthesized by Invitrogen (Carlsbad, CA, USA). After the PCR procedure, products were purified using the QiaQuick PCR Purification Kit (Qiagen) to remove unwanted constituents such as primer-dimers. All sequencing was performed by the UCLA Genotyping and Sequencing Core utilizing a 3730 capillary automated sequencer (Applied Biosystems, Foster City, CA, USA) using the forward primer for each product. Sequences were analyzed using the Applied Biosystems System Scanner Software and compared to wild type sequences obtained from the NCBI Entrez Gene database (Bethesda, MD, USA). For any samples presenting a genetic alteration in the target region, the PCR procedure was repeated and sequenced using both the forward and reverse primers for confirmation.

### FISH

Copy number of the EGFR gene was assessed using FISH in eighteen HNSCC cell lines. Briefly, cells in culture were treated with 0.05 g/mL colcemid (Sigma-Aldrich, St. Louis, MO, USA) for 24 hours to arrest cells in metaphase before harvesting with trypsin and fixed in a 3∶1 methanol:acetic acid solution. Preparation of samples, hybridization and microscopy were performed using previously established methods [28]. EGFR SpectrumOrange and CEP7 SpectrumGreen probes were used (Abbot Laboratories, Abbot Park, IL, USA), and samples were counterstained with 4′,6-diamidino-2-phenylin-dole (DAPI).

### Western Blots

Cultured cells in log-phase growth were treated with 100 nM dacomitinib, 100 nM erlotinib or 100 ug/mL cetuximab for one hour, with or without EGF stimulation (10 ng/mL). The plates were then washed twice with ice cold PBS, lysed and harvested using mild lysis buffer. Lysates were centrifuged at 10,000 RPM at 4C for 10 minutes to clear insoluble material and the resulting supernatant was collected and quantified using a bicinchoninic acid assay (Pierce Biochemicals, Rockford, IL, USA). Protein was resolved by SDS-PAGE and transferred to nitrocellulose membranes (Invitrogen). Anti-phospho-Akt (Ser-473), anti-total Akt, anti-phospho-ERK1/2 (T202/Y204), anti-total ERK1/2 and alpha-Tubulin antibodies were obtained from Cell Signaling Technologies (Danvers, MA, USA). Anti-phospho-EGFR (Y1068) antibody was obtained from Abcam (Cambridge, MA, USA). Anti-total EGFR was obtained from Santa Cruz Biotechnology (Santa Cruz, CA, USA). Western blots were quantified using ImageJ software.

### Cell Cycle and Apoptosis Analysis

The effects of dacomitinib and cetuximab on the cell cycle were investigated using Nim-DAPI staining (NPE Systems, Pembroke Pines, FL, USA). Cells were plated evenly in control and experimental wells and treated 24 hours later with 100 nM dacomitinib or 100 ug/mL cetuximab for five days. After aspirating media, cells were washed with PBS, released with trypsin and centrifuged at 3000 rpm for 5 minutes. The supernatant was removed and 100 uL of the Nim-DAPI solution was added. The solution was gently vortexed and allowed to incubate at room temperature for five minutes before analysis with UV using a Cell Lab Quanta SC flow cytometer (Beckman Coulter, Brea, California, USA).

Apoptosis assays were performed using an Annexin V-FITC apoptosis detection kit (MBL, Woburn, MA) and flow cytometry. Cells were plated and treated as for cell cycle studies and exposed to no drug, 100 nM dacomitinib or 100 ug/mL cetuximab for five days. After incubation cells were processed as directed in the kit and analyzed using a FITC signal detector and propidium iodide (PI) detector using a Cell Lab Quanta SC flow cytometer.
